# The complete mitochondrial genome sequence of *Lactarius trivialis* (Russulalles, Basidiomycota)

**DOI:** 10.1080/23802359.2020.1765217

**Published:** 2020-05-13

**Authors:** Shi-Cheng Shao, Yan Luo, Wen-Bin Yu

**Affiliations:** a Gardening and Horticulture Department, Xishuangbanna Tropical Botanical Garden, Chinese Academy of Sciences, Mengla, China; b Center for Integrative Conservation, Xishuangbanna Tropical Botanical Garden, Chinese Academy of Sciences, Mengla, China; c Center of Conservation Biology, Core Botanical Gardens, Chinese Academy of Sciences, Mengla, China

**Keywords:** *Lactarius trivialis*, mitochondrial genome, phylogenetic analysis, Russulalles

## Abstract

*Lactarius trivialis* is a very common species and widely distributes in north temperate. In this study, a complete mitogenome of *L. trivialis* was assembled and annotated. The whole mitogenome of *L. trivialis* was a circular molecule with 42,366 bp in length, encoding 44 genes as follows: 19 coding genes, two rRNAs, and 23 tRNAs. The contents of four bases in mtDNA were A (39.65%), T (38.82%), C (11.30%), and G (21.53%), respectively. Phylogenetic analysis recovered that it is nested with other *Lactarius* spp. in the order Russulalles.

As a large genus in Agaricoid taxa, *Lactarius* Pers. (Russulaceae) has high biodiversity with more than 600 species and form ectomycorrhiza (Buyck et al. 2010, 2018). *Lactarius* spp. were divided into six subgenera based on morphological evidences (Buyck et al. 2018). However, molecular phylogenies did not support morphological classifications (Buyck et al. 2018). Additional molecular marks are needed for species phylogenetic analyses and evolutionary analyses. The target species, *Lactarius trivialis*, belongings to subgenus *Piperites* (Fr. ex J. Kickx f.) Kauffman, and it is a very common species with a wide distribution in north temperate. It can be easily identified by its characteristics in robust fruit bodies, zonate pileus without strigose and color change with aging, and smooth stipe (Lee et al. 2019). In this study, a complete mitochondrial genome of *L. trivialis* was reported, which will provide genomic data for phylogenetic and evolutionary investigations of *Lactarius* and Russulaceae.

The sample of *L. trivialis* was bought in Yunnan Mushuihua wild edible mushroom market in Kunming, China (25°0′N, 102°43’E). Total DNA was stored in Yunnan Agricultural University, Yunnan, China with voucher no. MG71. The raw data of *L. trivialis* (Mycobank: 201199) was retrieved from NCBI (SRR5803928, https://trace.ncbi.nlm.nih.gov/Traces/sra/?run=SRR5803928), then *de novo* assembled to a complete and circular mitogenome using GetOrganelle (Jin et al. 2018). Gene annotation was accomplished using The MFannot tool (Beck and Lang 2010) (http://megasun.bch.umontreal.ca/cgi-bin/mfannot/mfannotInterface.pl) and manually checked in Geneious Primer 2020 (Biomatters, New Zealand). Sequences of Coding regions (CDS) and rRNAs were extracted, aligned, and concatenated using Geneious for phylogenomic analyses. Twenty species from Russulaceae, Boletales, and Agaricales were sampled, and *Ganoderma lucidum* (Curtis) P. Karst was selected as an outgroup. The best-fit model GTR + F+R5 using the Akaike information criterion (AIC) was applied to read the best model to construct Maximum-likelihood (ML) trees using IQ-TREE 1.6 (Lam-Tung et al. 2015).

The mitochondrial genome sequence of *L. trivialis* (GenBank accession MT267529) is a circular molecule with 42,366 bp in length, in which the contents of four bases were A (39.65%), T (38.82%), C (11.30%), and G (21.53%), respectively. The mitogenome encoded 44 genes as follows: 19 CDSs, two rRNAs, and 23 tRNAs. Typical ATG was used as the initiator codon and typical TAA as the terminator codon in all the 15 protein-coding genes (atp6, atp8, atp9, cob, cox1, cox2, cox3, nad1, nad2, nad3, nad4, nad4L, nad5, nad6, and rps3). The gene order and organization of the *L. trivialis* are consistent with congener species in *Lactarius* (Li, Ren et al., 2019; Li, Wang et al., 2019).

Phylogenomic analysis showed that *L. trivialis* clustered with other species of *Lactarius* spp. as a sister subclade to the subclade formed by *Russula* spp. (Figure 1), both which belonged to Russulalles. This newly reported mitchondrional genome of *L. trivialis* provide important genomic information for species evolution and phylogenetic analyses of *Lactarius* and Russulaceae.

**Figure 1.**
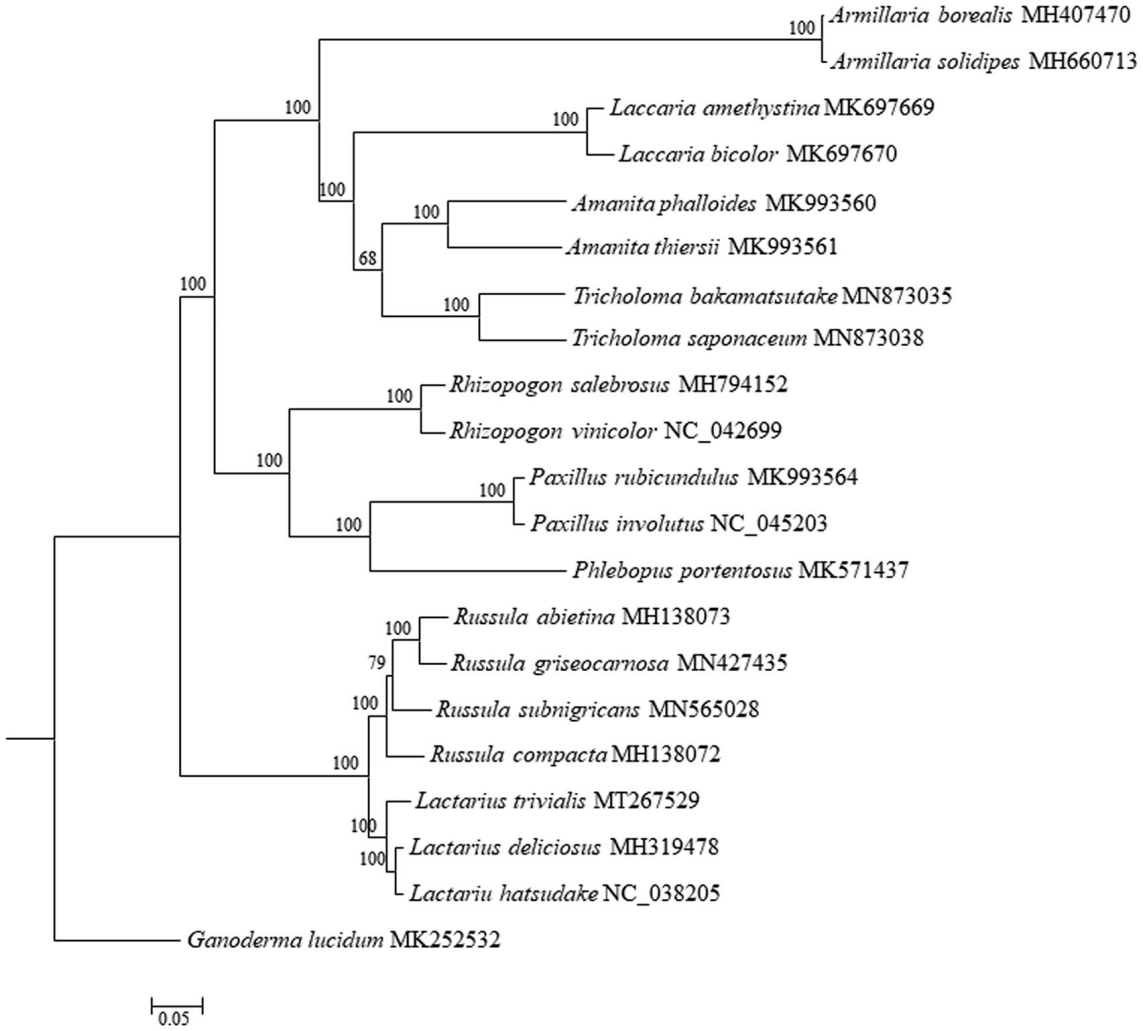
Phylogenetic analysis of *Lactarius trivialis* using maximum-likelihood of 15 protein-coding genes and two rRNA genes from 20 species from Russulaceae, Boletales, and Agaricales, and *Ganoderma lucidum* as outgroup. The bootstrap values were indicated close to the tree branches and each tip was followed by species name and accession number of GenBank.

## Data Availability

The data that support the findings of this study are available in GenBank with accession number MT267529. These data of *Lactarius trivialis* were derived from the following resources available in the public domain: NCBI Sequence Read Archive under accession number SRR5803928 (https://trace.ncbi.nlm.nih.gov/Traces/sra/?run=SRR5803928).
